# Editorial: Advanced veterinary topics in elasmobranchs

**DOI:** 10.3389/fvets.2025.1600135

**Published:** 2025-04-22

**Authors:** Natalie D. Mylniczenko

**Affiliations:** Disney's Animals, Science and Environment, Animal Programs, Lake Buena Vista, FL, United States

**Keywords:** shark, ray, veterinary, medicine, imaging, surgery

While sharks and rays are extremely popular fishes in aquariums, published elasmobranch veterinary medicine literature remains sparse (1–7). This Research Topic's articles pull together multiple disciplines to examine many important aspects of elasmobranch medicine that deserve greater attention.

Surgical articles detail procedures addressing ereproductive health in two of the most popular collected elasmobranch species. A case study on ovariectomy in the southern ray provides a basis for contraception and potentially resolving common reproductive disease issues. A study on Caesarian section in the cownose ray may help improve reproductive outcomes in other animals.

Two articles synergistically focus on the health and diseases of the inner ear. A vivid pictorial description of chondrocranial anatomy highlights normal inner ear anatomy and contrasts it with three large disease cases. Then a case series from multiple institutions describes diseases, diagnostics, and treatments of the endolymphatic system of the inner ear for the first time (Greene et al.).

Multiple articles present important diagnostics. Butyrate, a known energy molecule in sharks and rays, was reliably evaluated using hand held point of care units (Dannemiller et al.). A traumatic stingray case receives extensive veterinary medical imaging. Ultrasound is used to identify embryo abnormalities in shark eggs (Adams et al.).

Clinical articles provide additional tools for the treatment of elasmobranch disease. A pharmacologic article identifies systemically appropriate doses of voriconazole in undulate rays (Cañizares-Cooz et al.). A large case series of varying levels of ocular parasitic infiltration of corneas in cownose rays presents treatment strategies and outcomes. A sand tiger article diagnoses adomavirus in skin lesions and documents full resolution of clinical signs.

Finally, an important evaluation of thyroid assays found that traditional hormone testing has limited value for goiter, but that iodine testing may be valuable for this in combination with ultrasound and some previously unreported conditions (Wheaton et al.).

Elasmobranch medicine is an under-served topic in aquarium care. This compilation of articles shares the important research of many veterinarians working to improve the health of elasmobranchs in captivity.
